# Prognostic Value of Charlson Comorbidity Index in Patients with COVID-19

**DOI:** 10.3390/tropicalmed11020049

**Published:** 2026-02-10

**Authors:** Iliyan Todorov, Margarita Gospodinova, Kalina Stoyanova

**Affiliations:** Department of Infectious Diseases, Parasitology and Dermatovenerology, Faculty of Medicine, Medical University of Varna, 9002 Varna, Bulgaria

**Keywords:** COVID-19, SARS-CoV-2, comorbidities, Charlson Comorbidity Index

## Abstract

COVID-19, caused by SARS-CoV-2, is a highly contagious disease with variable clinical presentation. Severe forms are more common in patients with pre-existing chronic conditions. The objective of this study is to evaluate the prognostic value of the Charlson Comorbidity Index (CCI) in relation to disease severity and outcome in hospitalized COVID-19 patients with comorbidities. A retrospective analysis was conducted on 558 patients, hospitalized at the Infectious Diseases Clinic of “St. Marina” University Hospital, Varna, Bulgaria, between March 2020 and March 2021. CCI score was calculated to estimate 10-year survival probabilities. Prevalent comorbidities were arterial hypertension (78.55%), type 2 diabetes (16.09%), and ischemic heart disease (5.82%). A higher number of comorbidities was associated with increased rates of bilateral pulmonary consolidation (χ^2^ = 6.63, *p* = 0.010), oxygen therapy needs (χ^2^ = 5.41, *p* = 0.020), and mortality (χ^2^ = 7.88, *p* = 0.005). Patients with higher CCI scores had worse outcomes. A CCI score above 5 was common among non-survivors and those with pulmonary consolidation and respiratory failure. The findings confirm that advanced age and multiple comorbidities are strong predictors of poor COVID-19 prognosis. Early CCI calculation at hospital admission would help identify high-risk patients and support timely, targeted medical interventions.

## 1. Introduction

An outbreak of pneumonia that rapidly progressed to acute respiratory failure and was frequently associated with a fatal outcome emerged in Wuhan, Hubei Province, China, at the end of 2019. Preliminary investigations were unable to identify a specific etiologic agent. However, subsequent analyses concluded that a novel coronavirus designated as severe acute respiratory syndrome coronavirus-2 (SARS-CoV-2) was the causative agent of a newly recognized infectious disease: coronavirus disease-2019 (COVID-19) [1]. By 5 May 2023, the World Health Organization declared that COVID-19 was no longer a Public Health Emergency of International Concern (PHEIC). Nevertheless, SARS-CoV-2 continues to circulate on a global scale, and COVID-19 remains a persistent public health challenge [2]. By May 2023, more than 765 million confirmed cases and over 7 million associated deaths had been reported worldwide [3]. The disease is transmitted via respiratory droplets. Due to the high genetic variability in the virus—resulting from the specific characteristics of its replication cycle—new variants have continued to emerge periodically, each with distinct epidemiological and clinical features [4,5]. Today, although the pandemic has officially ended, SARS-CoV-2 continues to circulate globally. Therefore, it is essential for countries worldwide to maintain systems for continuous surveillance and to be prepared to implement timely public health responses in the event of a resurgence [6].

The clinical manifestation of COVID-19 varies and over 80% of patients experiencing a mild form that is characterized by influenza-like symptoms [7]. Severe disease, characterized by respiratory failure and the need for invasive mechanical ventilation, occurs more frequently in patients at the extreme ends of the age spectrum and in individuals with a compromised premorbid status. A multitude of comorbidities have been identified as factors that can adversely affect an individual’s prognoses. Among these, arterial hypertension, type 2 diabetes mellitus, ischemic heart disease, heart failure, chronic obstructive pulmonary disease, cerebrovascular diseases, malignancies, and chronic renal failure have been particularly noteworthy [8].

In this context, analyzing the impact of comorbidity on the prognosis of COVID-19 is essential for the timely identification of patients at high risk of complications and mortality. Various prognostic tools are employed for this purpose, including the Charlson Comorbidity Index (CCI) [9,10]. This indicator is relatively easy to calculate, and its strong prognostic value has been demonstrated across multiple diseases [11]. However, up to date, the CCI remains relatively underexplored and underutilized as a tool for early prognostic assessment in patients with COVID-19 and comorbidities.

The objective of this retrospective study is to evaluate the prognostic value of the Charlson Comorbidity Index (CCI) in relation to the clinical severity and early outcomes of COVID-19 specifically in hospitalized patients with at least one documented chronic comorbid condition. By focusing exclusively on this population, this study aims to provide insights into risk stratification at hospital admission while acknowledging limitations in generalizability to broader patient groups or vaccinated populations. Beyond its immediate clinical relevance, the present study contributes to the growing body of evidence necessary for retrospective validation, cross-country comparison, and reproducibility of prognostic models developed during the COVID-19 pandemic.

## 2. Materials and Methods

A retrospective analysis was conducted on the medical records of 558 patients diagnosed with COVID-19, hospitalized at the Infectious Diseases Clinic of “St. Marina” University Hospital, Varna, Bulgaria, between March 2020 and March 2021. Inclusion criteria comprised hospitalized adult patients with a complicated premorbid status defined as the presence of at least one documented chronic non-infectious comorbid condition, such as cardiovascular, metabolic, pulmonary, renal, neurological, oncological disorders or other chronic systemic conditions prior to SARS-CoV-2 infection. Written informed consent for hospitalization stay was obtained in advance, and confidentiality of personal data was ensured in accordance with national regulatory requirements. Patients treated on an outpatient basis, and hospitalized patients with an uncomplicated premorbid status were excluded from the analysis. All patients encompassed in this study had not received a vaccination, as these hospitalizations occurred prior to the initiation of the national vaccination campaign against the SARS-CoV-2 in Bulgaria.

The COVID-19 diagnosis was established based on a positive rapid antigen test performed triage office and subsequently confirmed by reverse-transcriptase polymerase-chain reaction (RT-PCR) of nasopharyngeal swabs at the Virology Laboratory of “St. Marina” University Hospital, Varna, Bulgaria. To determine the clinical severity of the disease, comprehensive clinical and laboratory investigations were performed, including the mandatory diagnostic minimum recommended by the National Institutes of Health [12], chest radiography, and when indicated, computed tomography (CT) of the lungs. Oxygen saturation was monitored daily through arterial blood gas analysis and multiple daily measurements using a portable pulse oximeter.

The Charlson Comorbidity Index was calculated for each patient at the time of hospital admission, based exclusively on documented pre-existing chronic conditions and prior to development of COVID-19-related complications (Table 1); version used in this study was assessed via the MD+ Calc platform [13]. Each comorbid condition contributed to a cumulative score corresponding to an estimated 10-year survival probability. A total score of 0 points correlates with a 98% estimated survival rate, one point with 96%, two points with 90%, three points with 77%, four points with 53%, five points with 21%, and scores of six or higher indicate the poorest prognosis, with only a 2% estimated chance of survival over the next 10 years. A subgroup analyses, including stratification by the number of comorbidities and CCI cut-offs, were introduced to provide a more detailed assessment of the association between comorbidity burden and disease severity. These analyses do not alter the original study design but offer enhanced insight into risk stratification.

The collected data is statistically analyzed using descriptive, variance, and correlation analyses within the SPSS software package, version 20.

## 3. Results

Of the 558 patients initially assessed, 66.85% (373/558) met the aforementioned inclusion criteria. The remaining 33.15% (185/558) of patients diagnosed with COVID-19 had no documented chronic non-infectious comorbidities prior to SARS-CoV-2 infection and were therefore classified as having an uncomplicated premorbid status and excluded from further analysis. The exclusion of patients without documented chronic comorbidities was intentional and methodologically justified. In this subgroup, no fatal outcomes were recorded, which precluded meaningful statistical comparisons regarding mortality risk. Inclusion of these patients would not have allowed for reliable assessment of potential non-linear relationships between comorbidity burden and outcomes due to the absence of events and the relatively small sample size. Moreover, hospitalized cohorts inherently reflect a selection toward more severe clinical presentations, as patients with mild disease and no comorbidities are frequently managed in outpatient settings.

Table 2 presents the distribution of the target patient group according to the number of comorbidities and key demographic characteristics (mean age and sex), as well as length of hospital stay and survivability.

The comorbidity profile of the studied patients was heterogeneous, encompassing a wide range of chronic non-communicable diseases affecting multiple organ systems. Cardiovascular and metabolic disorders predominated, with arterial hypertension being the most prevalent comorbidity, observed in 78.55% (293/373) of patients, followed by type 2 diabetes mellitus in 16.09% (60/373) and ischemic heart disease in 15.82% (59/373). Chronic pulmonary diseases were less frequently represented, including bronchial asthma in 4.56% (17/373) and chronic obstructive pulmonary disease in 4.29% (16/373) of cases. Other comorbid conditions included cerebrovascular disease, chronic kidney disease, malignancies, and chronic liver disease, each contributing to the cumulative Charlson Comorbidity Index score.

With regard to the clinical progression of COVID-19 in patients with preexisting medical conditions, the present study identified no statistically significant correlation between the prevalence of comorbidities and the incidence of lower respiratory tract involvement: 63.69% (107/168) in patients with one chronic disease, 65.38% (51/78) with two, and 65.40% (83/127) in individuals with three or more conditions (χ^2^ = 0.45, *p* = 0.800) (Table 3). Nevertheless, patients with extensive and diverse comorbidity profiles exhibited a higher propensity to develop more severe forms of pneumonia with progressive respiratory failure. A higher number of comorbidities was found to be significantly associated with increasing rates of bilateral pulmonary consolidation (χ^2^ = 6.63, *p* = 0.010), greater need for supplemental oxygen (χ^2^ = 5.41, *p* = 0.020), and increased risk of mortality (χ^2^ = 7.88, *p* = 0.005).

According to the CCI results calculated individually for each patient included in this study (Table 4), the highest estimated 10-year survival probability was observed in the group with only one comorbid condition (mean CCI score 1.54 ± 1.25), corresponding to a 90–96% survival rate. Patients with two comorbidities had a significantly higher mean CCI score of 2.58 ± 1.43 (77–90% 10-year survival), while those with three or more comorbidities showed a mean score of 4.08 ± 1.75, associated with 53% survival probability.

Beyond its relevance to the long-term prognosis, the CCI also demonstrated a relationship with early outcomes of the disease. Among patients with two, three, or more comorbidities who died during hospitalization, the total CCI score exceeded five points.

The findings further indicate a statistically significant association between higher CCI scores and pneumonia severity in the studied cohort. A total score below four was documented in patients with unilateral or bilateral patchy infiltrates in the lung parenchyma who did not require supplemental oxygen therapy. Conversely, among patients exhibiting varying degrees of respiratory failure with radiographic evidence of pulmonary consolidation (86/241), the CCI was significantly higher (*p* < 0.050), with scores exceeding 5 points in 35.68% of cases. When comparing survivors and non-survivors within the group of patients with two, three or more comorbidities, survivors had a mean CCI score of, respectively, 2.58 ± 1.43 and 4.08 ± 1.75, while non-survivors had a significantly higher mean score of 4 and 5.11 ± 2.28 (*p* = 0.032). This difference supports the hypothesis that a total CCI score above five points may serve as a clinically relevant cut-off for identifying patients at increased risk of early mortality during hospitalization due to COVID-19.

## 4. Discussion

The Charlson Comorbidity Index is a relatively simple and easy-to-use tool that can be used to estimate the 10-year survival probability of patients with various comorbid conditions across different age groups [9]. The model has been extensively studied by numerous authors, and increasingly, recent literature reflects its application in patients with COVID-19 [10,11,14,15,16,17]. Recent studies have indicated a correlation between elevated CCI scores and more severe clinical courses, increased mortality, and poorer long-term prognosis in cases of SARS-CoV-2 infection in the context of comorbidity [15,16]. Accordingly, the CCI is being used with greater frequency in clinical practice as a screening instrument to assist healthcare professionals in making informed decisions regarding the treatment and monitoring of high-risk patients [17].

The association between comorbidity burden and adverse COVID-19 outcomes has been previously explored in multiple cohorts. Earlier studies have demonstrated that higher CCI scores are linked to increased disease severity, need for intensive care, and mortality [10,14,16]. The present study confirms these findings in an Eastern European population hospitalized during the early pandemic phase and further refines the clinical interpretation of CCI by identifying a total score above five points as a potential threshold for early in-hospital mortality within this specific population, and the findings should not be extrapolated to all COVID-19 patients or later pandemic waves. In contrast to several prior analyses that combined vaccinated and unvaccinated individuals or spanned multiple variant waves [3.4.6], our cohort reflects a relatively homogeneous epidemiological context, thereby reducing confounding related to immunity status and variant-specific virulence.

Analysis of the data presented in Table 4 reveals that the long-term prognosis of COVID-19 in patients with comorbidities largely depends on the nature, type, and number of accompanying chronic conditions. These findings are consistent with those reported by other authors [8,18]. In our cohort, the mean CCI score in the group of patients with one comorbid condition was 1.54 ± 1.25 points, corresponding to a 96% estimated 10-year survival probability. Prognosis was significantly worse in the second and third subgroups, where the estimated risk of mortality within this timeframe was 77% and 53%, respectively. Intragroup comparisons demonstrated statistically significant differences (*p* < 0.050), confirming that the CCI plays a crucial role in clinical practice for assessing the long-term prognosis of patients with COVID-19 and comorbidities. The greater the number of registered comorbidities, the higher the likelihood of mortality within the subsequent 10 years. A 2020 study by Kuswardhani et al. [10] reported similar findings, indicating that higher CCI scores are associated both with more severe clinical courses of COVID-19 and with increased mortality in the studied patient population.

A statistically significant difference in mean CCI scores was observed among patients with two, three, or more chronic non-infectious diseases who died early during COVID-19–4 versus 5.11 ± 2.28 (*p* < 0.050). Therefore, a total CCI score above 5 could be considered a cut-off for high risk of early mortality, necessitating optimization and intensification of diagnostic and therapeutic management, as well as immediate consultation with specialists pertinent to the specific comorbid conditions. As shown in Table 4, nine out of 127 patients with three or more comorbidities died during hospitalization. According to Comoglu et al. [15], the risk of early fatal outcome increases by 2.5% for each additional point added to the total CCI score. Furthermore, this study emphasizes the significance of this scoring system in clinical practice as a mortality predictor. A score of four or higher has a capability to predict mortality with 87.2% sensitivity [15].

Beyond its immediate bedside applicability, the timely calculation of the CCI provides insight into the mechanisms linking multimorbidity with adverse COVID-19 outcomes. This necessitates optimizing and intensifying diagnostic and therapeutic algorithms and conducting timely consultations with various specialists, according to the profile of comorbidities. Such an approach is instrumental in facilitating the timely identification of complications that have the potential to influence not only the clinical progression of the disease but also the subsequent outcomes and the overall prognosis. From a broader perspective, these findings may also contribute to explaining population-level mortality patterns observed in certain regions, including potential post-pandemic mortality displacement effects.

It should be noted that the present study reflects early pandemic conditions, with cases predominantly occurring during periods dominated by the ancestral SARS-CoV-2 strain and the Alpha (B.1.1.7) variant. This phase of the pandemic was characterized by a largely unvaccinated population and evolving clinical management strategies. Subsequent studies have consistently demonstrated that later variants, particularly Omicron, in the context of widespread vaccination and hybrid immunity, are associated with substantially lower risks of severe disease and mortality compared to the ancestral strain and Alpha variant. However, despite these shifts in absolute risk, comorbidity burden has remained a robust and independent predictor of adverse outcomes across variant waves, supporting the continued relevance of comorbidity-based prognostic tools such as the CCI.

Patients from Eastern European populations, including Bulgaria, often present with a distinct metabolic and cardiovascular profile, characterized by higher prevalence of dyslipidemia, insulin resistance, obesity, and tobacco use. Collectively, these components may amplify the risk of severe outcomes from COVID-19 [19]. Furthermore, lifestyle factors such as smoking, alcohol consumption, diet, and the duration or chronicity of comorbid conditions have been identified as significant confounders, with the capacity to influence both the baseline health status and the trajectory of SARS-CoV-2 infection (e.g., healthier lifestyle patterns have been associated with lower risk of severe COVID-19 outcomes) [20]. Interestingly, several studies have reported a paradoxically lower risk of severe COVID-19 among patients with asthma or allergic diseases. These findings suggest a potential protective effect, possibly related to altered immune responses or regular use of inhaled corticosteroids, highlighting the complexity of interactions between specific comorbidities and COVID-19 severity [21,22,23,24].

## 5. Limitations

This study has several limitations that should be acknowledged. Its retrospective, single-center design may limit the generalizability of the findings to other populations and healthcare settings. The relatively limited number of deaths imposes constraints on the robustness of mortality-related analyses. Furthermore, potential confounding factors such as body mass index, smoking status, alcohol consumption, baseline disease severity, and duration of comorbid conditions were not consistently available and could not be adjusted for in the analysis. The study population reflects early pandemic conditions in a predominantly unvaccinated cohort, without variant-specific or vaccination-related stratification. It is imperative that these limitations be taken into consideration during the interpretation of the results and future multi-centre prospective studies are warranted to further validate the prognostic value of the CCI in cases of COVID-19.

## 6. Conclusions

In hospitalized patients with COVID-19 and documented chronic comorbidities, advanced age and the presence of multiple comorbidities were the strongest predictors of adverse outcomes. Arterial hypertension, type 2 diabetes mellitus, and ischemic heart disease were particularly significant. The CCI was higher in patients with severe disease, and elevated scores correlated with increased risk of early in-hospital mortality in this Bulgarian cohort during the early pandemic phase of the COVID-19. Calculating the CCI at the time of hospital admission can facilitate the early identification of high-risk patients and provides a robust framework for understanding the interaction between multimorbidity and COVID-19 severity, particularly in the context of future retrospective or comparative analyses.

## Figures and Tables

**Table 1:** Calculation of Charlson Comorbidity index (CCI).

Components	Score	
	None	Yes
Age <50 50–59 60–69 70–79 ≥80	0 +1 +2 +3 +4	
Myocardial infarction History of definite or probable myocardial infarction (EKG changes and/or enzyme changes)	0	+1
Congestive heart failure Exertional or paroxysmal nocturnal dyspnoea and has responded to digitalis, diuretics, or afterload reducing agents	0	+1
Peripheral vascular disease Intermittent claudication or past bypass for chronic arterial insufficiency, history of gangrene or acute arterial insufficiency, or untreated thoracic or abdominal aneurysm (≥6 cm)	0	+1
Cerebrovascular accident/Transient ischemic attack History of a cerebrovascular accident with minor or no residua and transient ischemic attacks	0	+1
Dementia Chronic cognitive deficit	0	+1
Chronic pulmonary disease	0	+1
Connective tissue disease	0	+1
Peptic ulcer disease Any history of treatment for ulcer disease or history of ulcer bleeding	0	+1
Liver disease Severe = cirrhosis and portal hypertension with variceal bleeding history, moderate = cirrhosis and portal hypertension but no variceal bleeding history, mild = chronic hepatitis (or cirrhosis without portal hypertension)	0	Mild +1 Moderate to severe +3
Diabetes mellitus	0	Uncomplicated +1 End-organ damage +2
Hemiplegia	0	+2
Moderate to severe chronic kidney disease Severe = on dialysis, status post kidney transplant, uraemia, moderate = creatinine >3 mg/dL (0.27 mmol/L)	0	+2
Solid tumour	0	Localized +2 Metastatic +6
Leukaemia	0	+2
Lymphoma	0	+2
Acquired Immunodeficiency Syndrome (AIDS)	0	+6

**Table 2:** Demographic and epidemiologic characteristics of patients with COVID-19 and comorbid conditions.

Index	Patients with One Comorbid Condition *n* = 168/373 (45.04%)	Patients with Two Comorbid Conditions *n* = 78/373 (20.91%)	Patients with Three and More Comorbid Conditions *n* = 127/373 (34.05%)
Mean age, years	56.86 ± 13.73	61.27 ± 11.28	66.05 ± 12.91
Sex, *n* (%): Male Female	89 (52.98) 79 (47.02)	44 (56.41) 34 (43.59)	55 (43.31) 72 (56.69)
Average length of hospital stay, (x ± SD), days	9.30 ± 2.99	9.28 ± 2.97	10.94 ± 4.98
Survivors, *n* (%)	168 (100)	77 (98.72)	118 (92.91)
Non-survivors, *n* (%)	0	1 (1.28)	9 (7.09)

**Table 3:** Demographic, clinic and laboratory characteristics of patients with pneumonia and comorbid conditions.

Indicators	Patients with Pneumonia and One Comorbid Condition *n* = 107/168 (63.69%)	Patients with Pneumonia and Two Comorbid Conditions *n* = 51/78 (65.38%)	Patients with Pneumonia and Three and More Comorbid Conditions *n* = 83/127 (65.40%)	
	Survivors *n* = 107 (100%)	Survivors *n* = 51 (100%)	Survivors *n* = 75/83 (90.40%)	Non-Survivors *n* = 8/83 (9.60%)
Sex, *n*, % Male Female	59 (55.14) 48 (44.86)	30 (58.82) 21 (41.18)	34 (45.30) 41 (54.70)	3 (37.50) 5 (62.50)
Mean age (x ± SD), years	58.06 ± 12.16	62.14 ± 10.72	67.81 ± 9.95	71.75 ± 19.02
Laboratory markers (x ± SD): CRP, mg/L Ferritin, ng/mL LDH, IU/L	93.01 ± 61.45 760.70 ± 1014.90 597.59 ± 205.34	106.0 ± 86.03 794.68 ± 728.58 668.98 ± 234.13	100.90 ± 110.52 621.91 ± 497.09 595.87 ± 185.21	233.67 ± 196.12 1384.03 ± 1758.14 957.25 ± 329.07
X-ray, *n* (%):				
Left patchy-linear opacities	17 (15.89)	4 (7.84)	5 (6.67)	0
Right patchy-linear opacities	33 (30.84)	4 (7.84)	9 (12.00)	0
Bilateral patchy-linear opacities	53 (49.53%)	17 (33.33%)	13 (17.33)	0
Right pulmonary consolidation	2 (1.87)	0	2 (2.67)	0
Left pulmonary consolidation	0	1 (1.96)	2 (2.67)	0
Bilateral pulmonary consolidation	2 (1.87)	25 (49.02)	44 (58.66)	8 (100)
SpO_2_ (x ± SD), %	95.33 ± 2.43	92.33 ± 6.01	90.75 ± 5.96	74.75 ± 6.96
Average length of hospital stay, (x ± SD), days	8.64 ± 2.43	11.55 ± 2.75	11.49 ± 3.50	13.38 ± 12.35

CRP—C-reactive protein, LDH—lactate dehydrogenase, SpO_2_—oxygenic saturation at atmosphere air.

**Table 4:** CCI in hospitalized patients with COVID-19 and comorbid conditions.

CCI, Points	Patients with One Comorbid Condition *n* = 168	Patients with Two Comorbid Conditions *n* = 78		Patients with Three and More Comorbid Conditions *n* = 127	
	Survivors *n* = 168 (100%)	Survivors *n* = 77 (97.77%)	Non-Survivors *n* = 1 (1.23%)	Survivors *n* = 118 (92.91%)	Non-Survivors *n* = 9 (7.09%)
0	45 (26.78%)	5 (6.50%)	0	2 (1.70%)	0
1	40 (23.81%)	13 (16.88%)	0	6 (5.10%)	1 (11.10%)
2	53 (31.55%)	21 (27.27%)	0	11 (9.30%)	1 (11.10%)
3	17 (10.12%)	17 (22.08%)	0	28 (23.70%)	0 (0.00%)
4	10 (5.95%)	12 (15.58%)	1 (100%)	26 (22.0%)	1 (11.10%)
5	3 (1.79%)	8 (10.39%)	0	20 (16.9%)	2 (22.20%)
6	0	1 (1.30%)	0	14 (11.90%)	0
7	0	0	0	8 (6.80%)	3 (33.30%)
8	0	0	0	2 (1.70%)	1 (11.10%)
9	0	0 that	0	1 (0.80%)	0
Mean, (x ± SD)	1.54 ± 1.25	2.58 ± 1.43	4	4.08 ± 1.75	5.11 ± 2.28
10 year survival, %	90–96%	77–90%	53%	53%	21%

## Data Availability

The data that support the findings of this study are available from the leading author, [I.T.], upon reasonable request.

## Abbreviations

The following abbreviations are used in this manuscript:

CCI	Charlson Comorbidity Index
COVID-19	Coronavirus disease—2019
CRP	C-reactive protein
LDH	Lactate dehydrogenase
SARS-CoV-2	Severe-acute respiratory syndrome coronavirus-2
SD	Standard deviation
SpO_2_	Oxygenic saturation at atmosphere air
